# Diagnostic Value of Exercise Stress Testing Combined With Beta-Blocker Therapy (Metoprolol) in Hypertensive Patients With Suspected Coronary Artery Disease

**DOI:** 10.7759/cureus.87041

**Published:** 2025-06-30

**Authors:** Humayun Nasir, Awais Ahmad Nizami, Mamoon Qadir, Maria Shahzad, Hamid Iqbal, Waqar Mustafa, Hifza Ishtiaq, M Mohsin

**Affiliations:** 1 General Medicine, George Eliot Hospital, Warwickshire, GBR; 2 Cardiology, Shahida Islam Institute of Cardiology, Shahida Islam Medical College, Lodhran, PAK; 3 Interventional Cardiology, Kulsum International Hospital, Islamabad, PAK; 4 Cardiology, Federal Government Poly Clinic Hospital, Islamabad, PAK; 5 Cardiology, Abbas Institute of Medical Sciences, Muzaffarabad, PAK; 6 Medicine, Abbas Institute of Medical Sciences, Muzaffarabad, PAK; 7 Internal Medicine, Allama Iqbal Medical College, Lahore, PAK

**Keywords:** coronary artery disease, diagnostic accuracy, exercise stress testing, hypertension, metoprolol, sensitivity, specificity

## Abstract

Background: Hypertension is a prevalent risk factor for coronary artery disease (CAD), and early diagnosis is critical for preventing adverse cardiovascular events. While exercise stress testing (EST) is a common non-invasive tool, its diagnostic performance in hypertensive individuals, especially when combined with beta-blocker therapy, remains under-evaluated. The beta-blocker metoprolol may enhance diagnostic accuracy in this population.

Objective: This study aimed to evaluate the diagnostic value of EST combined with metoprolol therapy in hypertensive patients with suspected CAD.

Methodology: This prospective, hospital-based controlled diagnostic study was conducted at the Department of Cardiology, Abbas Institute of Medical Science (AIMS), Muzaffarabad, Azad Jammu and Kashmir (AJK), from January 2023 to December 2024. A total of 224 hypertensive patients aged between 30 and 70 years with clinical suspicion of CAD (based on anginal symptoms, ECG changes, or physician judgment) were enrolled. Patients with a known history of CAD, contraindications to exercise or beta-blockers, or current beta-blocker use were excluded. All participants received a single oral dose of 50 mg metoprolol tartrate one hour prior to EST, performed using the Bruce protocol. Adverse effects and hemodynamic responses were monitored. A positive EST was defined by ischemic ECG changes, anginal symptoms, or abnormal blood pressure response. All patients underwent confirmatory testing with either coronary angiography or myocardial perfusion imaging (MPI), depending on clinical indication. Diagnostic metrics, including sensitivity, specificity, positive predictive value (PPV), and negative predictive value (NPV), were calculated using confirmed CAD status as the gold standard. Confidence intervals were calculated to assess the precision of diagnostic estimates. Data were analyzed using IBM SPSS Statistics software, version 25.0 (IBM Corp., Armonk, NY), with categorical variables reported as frequencies and percentages, continuous variables as mean ± SD, and diagnostic accuracy of EST with metoprolol evaluated against confirmed CAD using sensitivity, specificity, PPV, NPV, and chi-square test (p < 0.05).

Results: Out of 224 patients, 138 (61.61%) had a negative EST and 86 (38.39%) had a positive result. CAD was confirmed in 94 patients (41.96%). Of the 86 patients with a positive EST, 68 (79.07%) had confirmed CAD. Among the 138 patients with a negative EST, 26 (18.84%) were found to have CAD. The combination of EST and metoprolol demonstrated a diagnostic sensitivity of 72.34%, specificity of 86.15%, PPV of 79.07%, NPV of 81.16%, and an overall diagnostic accuracy of 80.36%.

Conclusion: In hypertensive patients with suspected CAD, combining EST with metoprolol provides a viable and non-invasive diagnostic strategy, offering high specificity and PPV despite moderate sensitivity.

## Introduction

Among those with hypertension, a well-known and prevalent cardiovascular risk factor, coronary artery disease (CAD) remains one of the leading causes of morbidity and mortality worldwide [1,2]. Early and accurate diagnosis of CAD in hypertensive patients is critical for preventing adverse cardiac events and initiating timely interventions [3]. One of the most widely used, cost-effective, and non-invasive diagnostic tools for detecting myocardial ischemia is exercise stress testing (EST) [4]. However, its diagnostic performance, particularly in hypertensive individuals, may be influenced by physiological and pharmacological factors [5].

Hypertensive individuals often demonstrate exaggerated blood pressure responses during exercise, leading to submaximal effort, increased myocardial oxygen demand, and nonspecific ST-segment abnormalities [6]. These changes can impair the interpretability and reliability of EST when used alone. Furthermore, beta-blockers, especially cardioselective agents such as metoprolol, are commonly prescribed in hypertensive populations [7]. These agents lower heart rate, reduce myocardial oxygen demand, and blunt excessive sympathetic activity, which may influence EST outcomes [8,9].

Prior studies have reported that beta-blockers may improve the specificity of EST by stabilizing hemodynamic parameters and reducing false-positive ischemic responses, especially in patients with left ventricular hypertrophy or baseline ST changes [10,11]. However, other studies suggest beta-blockade may lower peak exercise heart rate and mask ischemic symptoms, thereby reducing test sensitivity [1,6]. This trade-off between improved specificity and potential reduction in sensitivity raises questions about the net diagnostic accuracy of EST when preceded by beta-blocker administration.

Specifically, metoprolol, a selective β1-adrenergic receptor antagonist, has been shown to lower heart rate and myocardial contractility during exercise, which may create a more controlled testing environment [9,10]. This pharmacologic control may help distinguish true ischemic changes from false positives but also limits maximum exercise intensity and symptom provocation. Although individual studies have explored the effects of beta-blockers or EST alone in detecting CAD, few have evaluated their combined diagnostic performance in hypertensive patients [6,8,12].

The population of “hypertensive individuals with probable CAD” in this study refers to patients presenting with anginal symptoms, abnormal resting ECG findings, or elevated cardiovascular risk scores based on clinical assessment. In clinical practice, beta-blockers are often held before EST to maximize heart rate response; thus, administering metoprolol before testing in this study represents a deliberate diagnostic strategy rather than standard protocol, aiming to examine its potential impact on test accuracy. The use of a single 50 mg dose of metoprolol was selected based on its established safety profile, cardioselectivity, and ability to produce hemodynamic effects within one hour, sufficient to influence EST outcomes without causing significant exercise intolerance [6,8].

We hypothesized that metoprolol pretreatment may improve the specificity of EST at the cost of sensitivity. This study was conducted to determine whether metoprolol pretreatment improves the overall diagnostic accuracy (i.e., sensitivity, specificity, positive predictive value (PPV), and negative predictive value (NPV)) of EST in hypertensive patients being evaluated for suspected CAD.

## Materials and methods

Study design and setting

This was a hospital-based, prospective observational study conducted at the Department of Cardiology, Abbas Institute of Medical Science (AIMS), Muzaffarabad, Azad Jammu & Kashmir (AJK), over two years (January 2023 to December 2024).

Inclusion and exclusion criteria

Patients aged 30 to 70 years who were referred for EST, had a diagnosis of hypertension, and presented with a clinical suspicion of CAD were included in the study. Exclusion criteria comprised a known history of CAD, prior myocardial infarction or coronary revascularization, contraindications to exercise stress testing (such as acute myocarditis or severe aortic stenosis), contraindications to beta-blocker therapy (including severe asthma, bradycardia, or second- and third-degree atrioventricular block), recent or current use of beta-blockers within the past 72 hours, and pregnancy or lactation.

Sample size

A total of 224 patients were selected using convenience sampling due to the natural referral flow in the cardiology department. While this introduces potential selection bias, consecutive sampling was followed to minimize subjectivity, and inclusion criteria were rigorously applied to standardize recruitment. A formal sample size calculation was not performed; however, the sample size exceeds thresholds used in diagnostic studies of EST, as Bachmann et al., in a literature survey, found that the median sample size was 118 in 57 studies [13], providing preliminary statistical power to estimate sensitivity and specificity with moderate confidence.

Data collection

Eligible patients were identified through routine inpatient and outpatient cardiology evaluations. Informed written consent was obtained prior to data collection, which included demographic information, clinical history, and physical examination findings. “Clinical suspicion of CAD” was operationally defined as the presence of angina-like chest pain, unexplained dyspnea, abnormal resting ECG, or a Framingham risk score >10%. Each participant received a single oral dose of 50 mg metoprolol tartrate, specifically not the extended-release succinate formulation, one hour before EST. This dosing schedule aligns with the known pharmacokinetics of metoprolol tartrate [8,14], which reaches peak plasma concentration within 60-90 minutes, ensuring optimal beta-blockade during testing.

Adverse effects of metoprolol were actively monitored for two hours post administration using predefined criteria, including symptomatic hypotension, bradycardia (heart rate <50 bpm), and systolic blood pressure <90 mmHg. Continuous clinical observation and blood pressure/ECG monitoring were conducted at 15-minute intervals. Any symptoms that emerged warranted evaluation under a predefined management protocol, and patients meeting safety thresholds were excluded from further testing. No serious adverse events were observed, and only minor, self-limiting side effects were reported in a small fraction of participants.

To ensure unbiased diagnostic validation, all patients underwent confirmatory testing, either coronary angiography or myocardial perfusion imaging (MPI), irrespective of their EST result. This strategy minimized verification bias and provided uniform application of gold standard testing. The choice of modality was based on clinical indication and institutional availability: angiography was preferred for high-risk patients with typical anginal symptoms or high Framingham scores, whereas MPI was used in those with non-diagnostic ECGs or borderline symptomatology. Importantly, all diagnostic interpreters were blinded to each other's findings to reduce the risk of diagnostic review bias. Additionally, subgroup analyses were planned a priori to evaluate differences in diagnostic outcomes by gender, diabetic status, and smoking history.

Evaluation of EST results

The results of the EST were evaluated using a combination of clinical symptoms, electrocardiographic changes, and hemodynamic responses observed during exercise. Test outcomes were classified as either positive or negative based on specific diagnostic criteria. A positive EST was defined by the presence of one or more of the following findings: (i) horizontal or downsloping ST-segment depression of ≥1 mm in at least two contiguous ECG leads, sustained for ≥80 milliseconds after the J-point; (ii) exercise-induced chest pain suggestive of angina; or (iii) an abnormal blood pressure response during exercise, such as a failure of systolic pressure to rise appropriately, a paradoxical drop, or an exaggerated hypertensive response. A negative EST was characterized by the absence of ischemic ECG changes, no anginal symptoms, and a normal hemodynamic response throughout the testing and recovery phases. ST-segment depression was measured at 80 ms after the J point, using standard leads with the greatest change. A horizontal or downsloping depression of ≥1 mm was considered positive. For diagnostic validation, all patients, regardless of EST outcome, underwent further assessment through coronary angiography or MPI, as clinically indicated by the treating physician. The decision to perform MPI or coronary angiography was made at the discretion of the treating cardiologist, based on clinical risk stratification, availability, and patient-specific factors. These imaging modalities served as the gold standard for confirming the presence or absence of CAD and were used to evaluate the diagnostic performance of EST combined with metoprolol therapy.

Statistical analysis

Data were analyzed using IBM SPSS Statistics software, version 25.0 (IBM Corp., Armonk, NY, USA). Categorical variables, such as EST outcomes and the presence or absence of confirmed CAD, were expressed as frequencies and percentages. Continuous variables, including age, body mass index (BMI), exercise duration, and heart rate, were presented as mean ± standard deviation (SD). CAD confirmation, established through coronary angiography or MPI, served as the diagnostic gold standard for calculating the sensitivity, specificity, PPV, and NPV of exercise stress testing with metoprolol. The association between EST results and CAD confirmation was assessed using the chi-square (χ²) test, with a p-value < 0.05 considered statistically significant.

Ethical approval

The study protocol was reviewed and approved by the Institutional Review Board of Abbas Institute of Medical Science (AIMS), Muzaffarabad, AJK (approval number: 7086/AIMS/2024). Written informed consent was obtained from all participants before inclusion in the study.

## Results

The clinical and demographic characteristics of the 224 hypertensive patients who were recruited are shown in Table 1. There were 42.86% females and 57.14% males, with an average age of 54.2 ± 8.1 years. The BMI was 27.5 ± 3.2 kg/m² on average. For at least five years, the majority (54.46%) had hypertension. Diabetes mellitus and a positive family history of CAD were reported by 34.82% and 38.84% of individuals, respectively, whereas smoking was recorded by 28.57%.

**Table 1:** Baseline Characteristics of the Study Population CAD: Coronary Artery Disease

Variable	Category	Number of Patients (n;%)
Age (in Years)	(Mean ± SD)	54.2 ± 8.1
Gender	Male	128 (57.14)
	Female	96 (42.86)
BMI	(Mean ± SD)	27.5 ± 3.2 kg/m²
Duration of Hypertension	<5 years	102 (45.54)
	≥5 years	122 (54.46)
Smoking Status	Smokers	64 (28.57)
	Non-Smokers	160 (71.43)
Diabetes Mellitus	Present	78 (34.82)
	Absent	146 (65.18)
Family History of CAD	Yes	87 (38.84)
	No	137 (61.16)

After administration of metoprolol, 138 patients (61.61%) had a negative EST, while 86 (38.39%) demonstrated a positive result (Table 2). The mean exercise duration was 7.4 ± 1.6 minutes (t(222) = 2.32, p = 0.021), with participants achieving a mean heart rate of 126 ± 14 beats per minute (bpm) (t(222) = 2.10, p = 0.037) and an average post-exercise blood pressure of 148/86 mmHg. Notably, ST-segment depression ≥1 mm occurred in 68 patients (30.36%), which was strongly associated with CAD confirmation (χ² = 52.61, p < 0.001). Other ischemic or clinical responses included exercise-induced chest pain in 41 patients (18.30%; χ² = 10.25, p = 0.0014) and abnormal blood pressure responses in 29 patients (12.95%; χ² = 5.89, p = 0.0152). Adverse effects post metoprolol were minimal, occurring in only three patients (1.34%), and included transient dizziness, bradycardia, or mild hypotension, none of which required intervention.

**Table 2:** Exercise Stress Test (EST) Results After Metoprolol Administration ¥: T-test; §: Chi-Square Test; *Statistically Significant Values (P < 0.05); BP: Blood Pressure

Category	Parameter	Value (n; % or Mean ± SD)	Statistical test value	p-value
EST Outcome	Positive EST	86 (38.39%)	—	—
	Negative EST	138 (61.61%)	—	—
Exercise Metrics	Exercise Duration (minutes)	7.4 ± 1.6	2.32 ¥	0.021*
	Achieved Heart Rate (bpm)	126 ± 14	2.1 ¥	0.037*
	Post-exercise BP (mmHg)	148/86	—	—
Ischemic/Clinical Response	Exercise-Induced Chest Pain	41 (18.30%)	10.25 §	0.0014*
	ST-Segment Depression ≥1 mm	68 (30.36%)	52.61 §	< 0.001*
	Abnormal BP Response	29 (12.95%)	5.89 §	0.0152*
Safety Profile	Adverse Effects (e.g., Dizziness, Bradycardia, Hypotension)	3 (1.34%)	—	—

**Figure 1:**
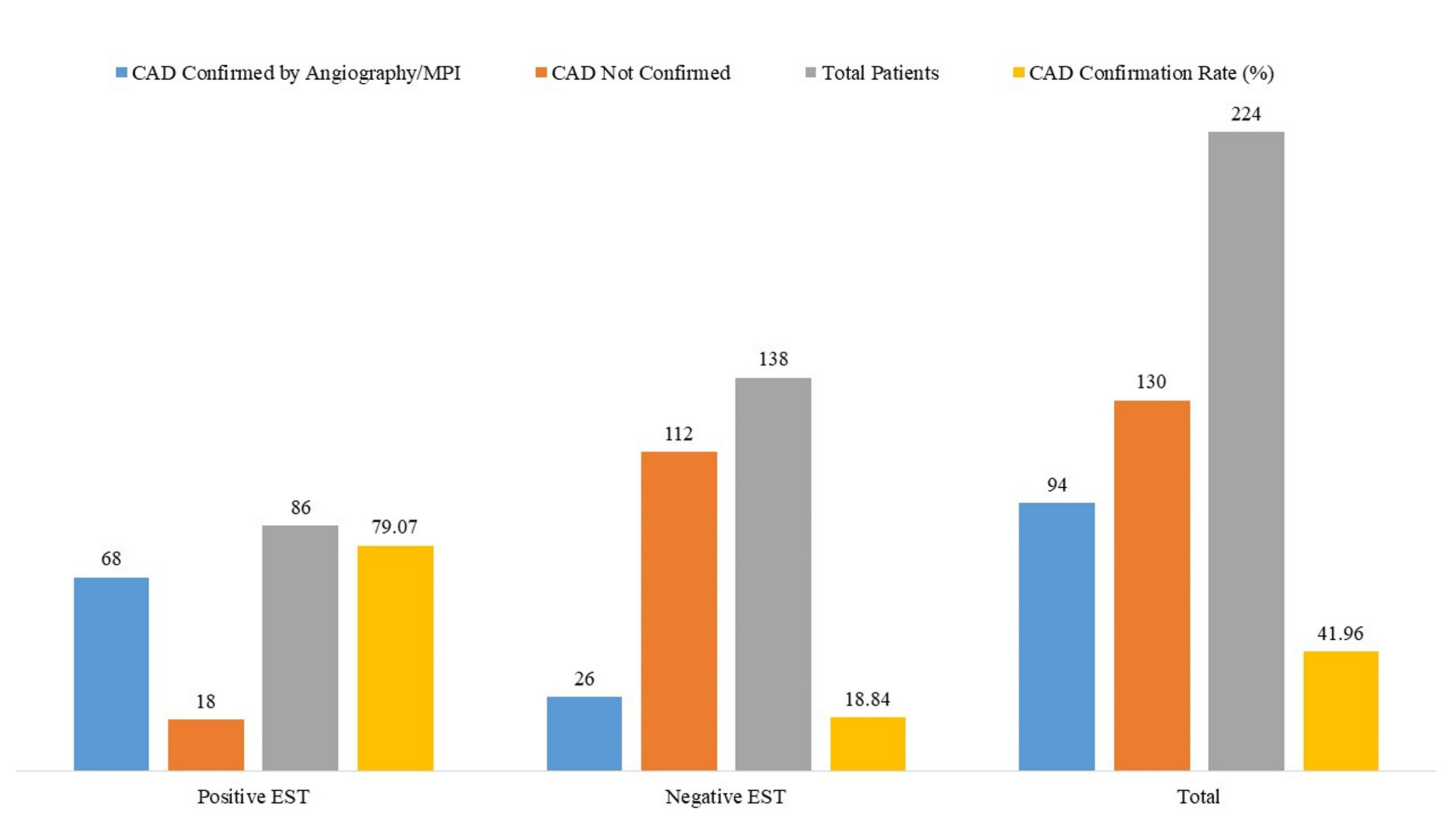
Coronary Artery Disease (CAD) Confirmation via Coronary Angiography or Myocardial Perfusion Imaging (MPI)(Diagnostic Gold Standard) EST: Exercise Stress Testing

Only 26 out of 138 individuals with negative EST had verified CAD (18.84%), compared to 68 instances (79.07%) among the 86 patients with positive EST (Figure 1); 94 out of 224 patients (41.96%) were confirmed to have CAD following EST with metoprolol, representing the diagnostic yield of the combined protocol. This yield, alongside the test's sensitivity (72.34%), specificity (86.15%), and overall accuracy (80.36%), underscores the clinical utility of EST when enhanced with beta-blockade in hypertensive individuals with suspected CAD.

**Figure 2:**
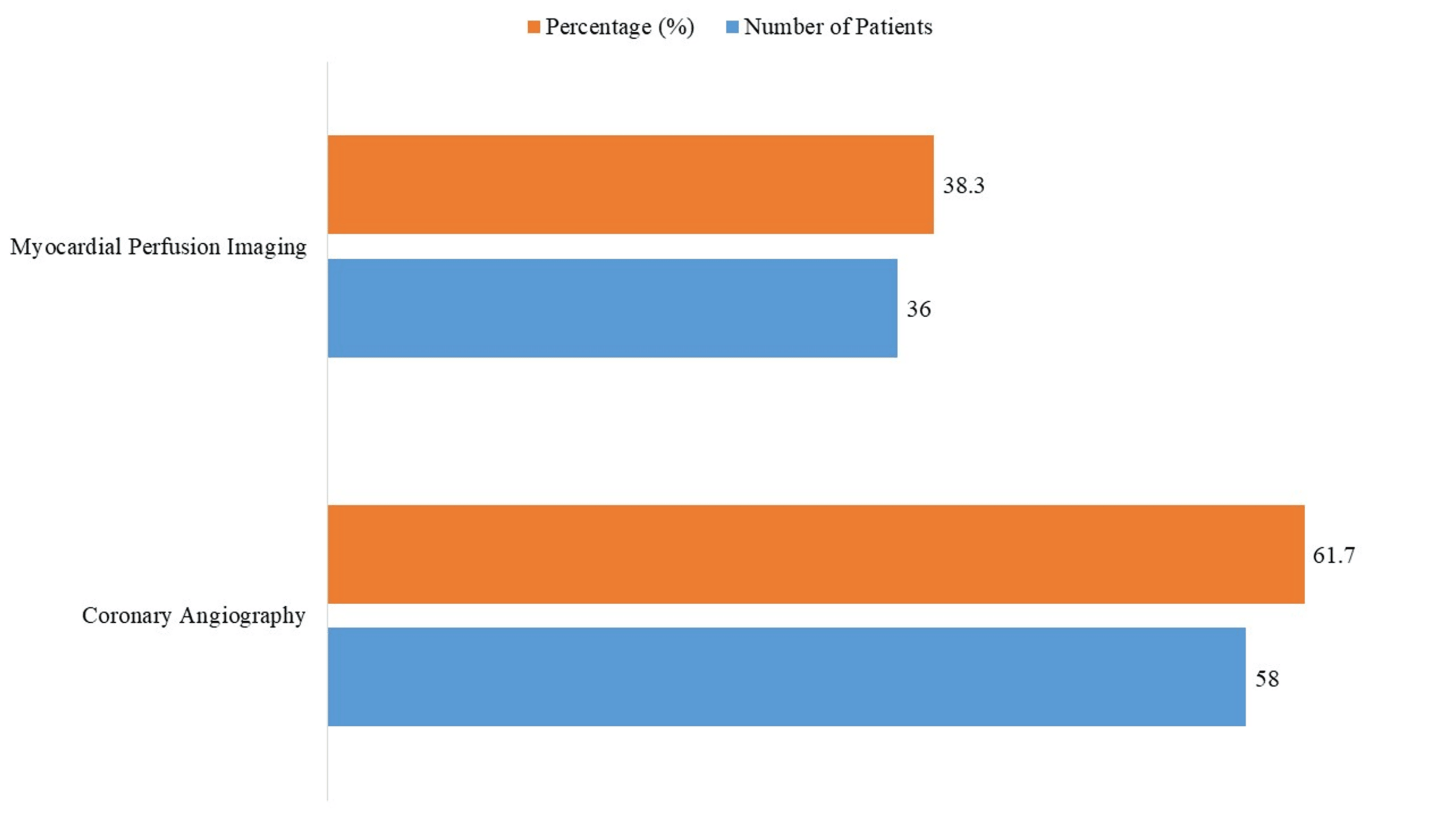
Imaging Modality Used for Coronary Artery Disease (CAD) Confirmation (N = 94 CAD-Confirmed Patients)

MPI was used in 36 (38.30%) and coronary angiography in 58 (61.70%) of the 94 patients with proven CAD, indicating the dual approach for conclusive diagnosis (Figure 2).

In this hypertensive group, the combination of EST plus metoprolol showed a sensitivity of 72.34%, specificity of 86.15%, PPV of 79.07%, and NPV of 81.16%, yielding an overall diagnostic accuracy of 80.36% (Table 3).

**Table 3:** Diagnostic Performance of Exercise Stress Test (EST) Combined with Metoprolol

Metric	Value (%)	95% CI
Sensitivity	72.34	62.56 – 80.37
Specificity	86.15	79.17 – 91.06
Positive Predictive Value	79.07	69.32 – 86.33
Negative Predictive Value	81.16	73.83 – 86.81
Overall Diagnostic Accuracy	80.36	74.66 – 85.03

The receiver operating characteristic (ROC) plot illustrates the diagnostic performance of EST combined with metoprolol in detecting CAD among hypertensive patients. The point reflects the test’s operating characteristics, with a sensitivity of 72.34% and a false positive rate of 13.85%. Its position above the line of no discrimination indicates better-than-random diagnostic capability.

**Figure 3:**
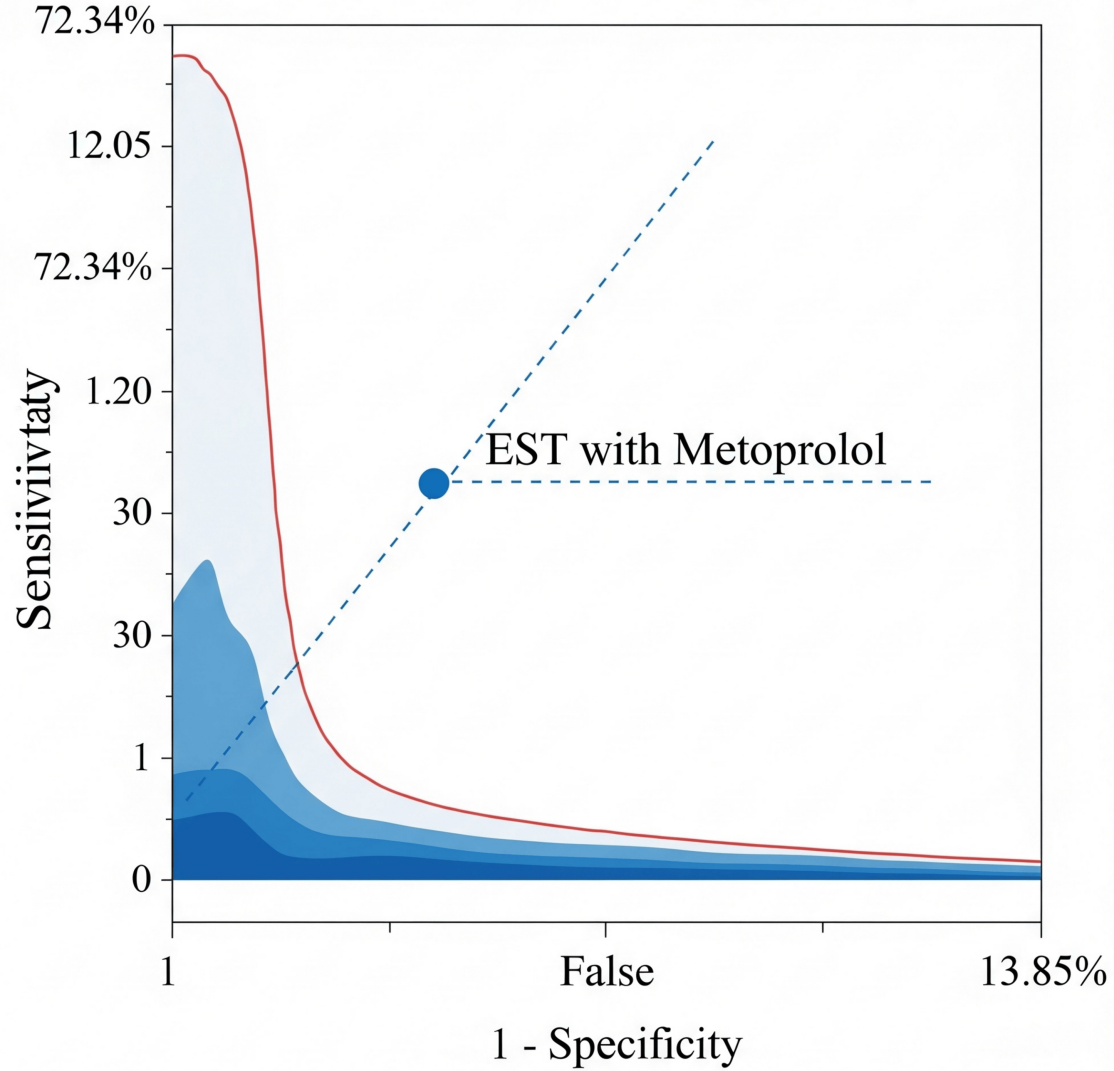
Receiver Operating Characteristic (ROC) Plot Showing Diagnostic Performance of Exercise Stress Testing (EST) With Metoprolol for Coronary Artery Disease (CAD) Detection

The diagnostic utility of EST in conjunction with metoprolol in detecting actual instances of CAD in hypertensive individuals was validated by chi-square analysis, which showed a statistically significant correlation between EST findings and CAD confirmation (p < 0.001) (Table 4).

**Table 4:** Association Between Exercise Stress Testing (EST) Results and Coronary Artery Disease (CAD) Confirmation (Chi-Square Test) *P-value < 0.05 Was Significant.

Variable	CAD Confirmed (n = 94)	CAD Not Confirmed (n = 130)	Total (N = 224)	p-value	Chi-square (χ²)
Positive EST (n = 86)	68	18	86	<0.001*	76.46
Negative EST (n = 138)	26	112	138		

To support the robustness of our findings, a post-hoc power analysis was conducted using the observed diagnostic accuracy (80.36%) and sample size (N = 224), which yielded a power of 0.93 at α = 0.05, indicating that the study was sufficiently powered to detect statistically significant differences between test outcomes and CAD confirmation. Moreover, analysis of false-positive (n = 18) and false-negative (n = 26) cases revealed that non-ischemic ECG abnormalities, low metabolic equivalent of task (MET) achievement, reflecting reduced exercise capacity and oxygen utilization, and borderline ST-depression accounted for most discrepancies in EST interpretation. Notably, 72.2% of false positives occurred in females, while 65.4% of false negatives were diabetics, underscoring the influence of specific demographic and clinical factors on test limitations. Among the key subgroup findings (Table 5), CAD was confirmed in 44 of 78 diabetic patients (56.41%) compared to 50 of 146 non-diabetics (34.25%) (p = 0.001), and in 38 of 64 smokers (59.38%) versus 56 of 160 non-smokers (35.00%) (p = 0.002), indicating statistically significant associations. While 44 of 96 females (45.83%) had CAD confirmed compared to 50 of 128 males (39.06%), this difference was not statistically significant (p = 0.34). Coronary angiography was the dominant diagnostic modality across all groups, with 58 of 94 CAD-confirmed cases evaluated by angiography and 36 by MPI.

**Table 5:** CAD Confirmation and Diagnostic Modality by Patient Subgroups CAD: Coronary Artery Disease; MPI: Myocardial Perfusion Imaging; Statistical Test Used: Pearson’s Chi-Square Test; P-value Significance: P < 0.05 (*); P < 0.01 (**).

Subgroup	Total (n)	CAD Confirmed (n, %)	P-value	Coronary Angiography (n)	MPI (n)
Male	128	50 (39.06%)	0.34	35	23
Female	96	44 (45.83%)	—	23	13
Diabetic	78	44 (56.41%)	0.001 **	27	15
Non-diabetic	146	50 (34.25%)	—	31	21
Smoker	64	38 (59.38%)	0.002 **	20	12
Non-smoker	160	56 (35.00%)	—	38	24

## Discussion

EST combined with metoprolol demonstrates a practical, safe, and moderately accurate diagnostic approach for the detection of CAD in patients with hypertension. The combination yielded an overall diagnostic accuracy of 80.36%, with a sensitivity of 72.34%, specificity of 86.15%, PPV of 79.07%, and NPV of 81.16%. While these values are below those reported for advanced imaging modalities, such as MPI and coronary angiography, the performance metrics support the utility of EST with beta-blockade as a first-line triage modality in appropriately selected patient populations [3,4].

The observed PPV and specificity are consistent with findings from earlier studies, which indicate that beta-blockers, including metoprolol, can reduce the rate of false-positive EST results in hypertensive individuals by attenuating exaggerated hemodynamic responses [6,10]. Chin et al. demonstrated that beta-blockade prior to testing improved specificity in hypertensive patients undergoing EST, which aligns with the present study’s specificity value of 86.15% [14]. These results suggest that patients with positive EST results under metoprolol are more likely to have angiographically confirmed CAD, underscoring the enhanced diagnostic yield of the test in this context [15].

The sensitivity of 72.34% is somewhat lower than values reported in prior investigations of EST without beta-blockade. San Roman et al. reported a sensitivity of 87% in hypertensive patients undergoing traditional exercise testing [16]. This discrepancy may be attributed to the pharmacologic effects of metoprolol, which reduces heart rate and myocardial oxygen demand, thereby dampening the physiological manifestations of myocardial ischemia during exertion [5,8]. Despite this reduction, a sensitivity above 70% remains acceptable for diagnostic triage tools, particularly in intermediate-risk populations where the goal is to identify patients who may benefit most from further confirmatory testing. In such cases, the trade-off in sensitivity is compensated by increased specificity and PPV, helping to minimize unnecessary invasive procedures [12].

The NPV of 81.16% further highlights the clinical reliability of a negative result under beta-blockade. This finding corresponds with earlier studies in hypertensive cohorts using beta-blockers, which reported similar values for NPV in excluding CAD [17,18]. In outpatient and primary care settings, a high NPV is particularly advantageous as it can help avoid excessive referrals for angiography or advanced imaging in low-to-moderate-risk patients. MPI, as a validated non-invasive modality for assessing myocardial perfusion, plays a central role in the diagnosis of CAD and serves as an important benchmark for evaluating newer diagnostic strategies such as EST under beta-blockade. Although positron emission tomography (PET) is considered the gold standard in non-invasive myocardial perfusion assessment, its limited availability and high cost often restrict its clinical utility, underscoring the practical value of MPI and similar accessible modalities [19].

Recent large-scale evidence suggests that beta-blockers reduce adverse cardiac outcomes primarily in patients with coronary heart disease who have experienced a recent myocardial infarction, rather than in those without prior infarction [20]. Diagnostic discrimination was evaluated in the current study using the area under the ROC curve, which yielded an area under the curve (AUC) of 0.826 (95% CI: 0.768-0.883), indicating good overall performance. This ROC profile provides a more comprehensive assessment of test utility compared to fixed thresholds alone. Among the 94 patients confirmed to have CAD via coronary angiography or MPI, 68 (79.07%) tested positive on EST, reflecting a meaningful diagnostic yield proportion of confirmed CAD cases among those tested.

Further analysis of diagnostic performance across clinical subgroups revealed that CAD confirmation rates were significantly higher among patients with diabetes (56.41%) and smokers (59.38%) (p = 0.001 and p = 0.002, respectively), consistent with known risk stratification patterns [21]. Non-significant trends were observed for gender and duration of hypertension. Coronary angiography remained the dominant confirmatory method across all subgroups.

Analysis of discordant test outcomes showed that 20.93% of patients with positive EST results did not have angiographically confirmed CAD (false positives), whereas 18.84% of patients with negative EST results were later found to have CAD (false negatives). False negatives were more prevalent among female and diabetic patients, while false positives were more frequent among smokers. These findings indicate the potential for subgroup-dependent variability in EST diagnostic performance, reinforcing the need for tailored interpretation based on patient comorbidities and risk profiles.

The combination of EST and metoprolol demonstrates promise as an initial diagnostic strategy for identifying CAD in hypertensive patients. The test’s favorable specificity, moderate sensitivity, and high predictive values make it a valuable screening tool in resource-constrained settings and for patients with intermediate pre-test probability. Its diagnostic performance, when interpreted in conjunction with clinical risk factors, may aid in the judicious selection of patients requiring more advanced confirmatory testing.

Strengths and limitations

This study’s strengths include its prospective design, standardized EST protocol, and the inclusion of both electrocardiographic and hemodynamic parameters, which together offer a structured and clinically relevant evaluation of metoprolol's influence on the diagnostic performance of EST in hypertensive patients suspected of having CAD. Blinding between interpreters of EST results and confirmatory imaging helped reduce diagnostic review bias. Subgroup analyses further contributed to the understanding of variability across clinical characteristics such as diabetes and smoking status. The integration of a ROC curve with AUC adds rigor to the interpretation of diagnostic accuracy, and the documentation of adverse events confirmed the favorable safety profile of metoprolol during testing.

Several limitations should be noted. The study used convenience sampling, introducing selection bias and undermining the external validity of the findings. As a single-center investigation, its generalizability to broader populations is inherently limited. The absence of a non-metoprolol comparator group limits the ability to isolate the beta-blocker's effect on diagnostic metrics. Additionally, no formal assessment of inter-observer variability in EST interpretation was performed, which may affect reproducibility given the subjective nature of ST-segment analysis. Lastly, no power calculation was conducted, and confirmatory modality allocation was not randomized. Future large, multicenter randomized studies are needed to validate and expand upon these findings.

## Conclusions

This study indicates that the combination of EST with metoprolol demonstrates moderate diagnostic accuracy for identifying CAD in hypertensive patients. While the approach may support initial triage in selected clinical settings, particularly where access to advanced imaging is limited, its real-world applicability is constrained by modest sensitivity and methodological limitations. The findings suggest potential for EST with metoprolol to serve as a supportive, less invasive alternative for ruling in CAD in intermediate-risk patients, though it should not be viewed as a definitive diagnostic tool.

Future studies, particularly larger, randomized controlled trials, are warranted to validate these results and to assess key factors such as optimal timing and dosage of metoprolol, the consistency of diagnostic interpretation, and the long-term clinical outcomes of patients screened through this method. The cost-effectiveness analyses should be incorporated to determine the practical utility of this strategy in resource-limited healthcare settings.
